# Acceptability and Willingness-to-Pay for a Hypothetical Ebola Virus Vaccine in Nigeria

**DOI:** 10.1371/journal.pntd.0003838

**Published:** 2015-06-15

**Authors:** Maduka Donatus Ughasoro, Dorothy Omono Esangbedo, Beckie Nnenna Tagbo, Ijeoma Chigozie Mejeha

**Affiliations:** 1 Department of Paediatrics, University of Nigeria, Enugu Campus, Enugu, Nigeria; 2 Paediatric Division, Providence Hospital, Ikoyi, Lagos, Nigeria; 3 Institute of Child Health/ Department of Paediatrics, University of Nigeria Teaching Hospital, Ituku/Ozalla, Enugu, Nigeria; 4 Department of Paediatrics, Federal Medical Centre, Umuahia, Abia State, Nigeria; Tulane School of Public Health and Tropical Medicine, UNITED STATES

## Abstract

**Background:**

Ebola virus disease is a highly virulent and transmissible disease. The largest recorded fatality from Ebola virus disease epidemic is ongoing in a few countries in West Africa, and this poses a health risk to the entire population of the world because arresting the transmission has been challenging. Vaccination is considered a key intervention that is capable of arresting further spread of the disease and preventing future outbreak. However, no vaccine has yet been approved for public use, although various recombinant vaccines are undergoing trials and approval for public use is imminent. Therefore, this study aimed to determine the acceptability of and willingness-to-pay for Ebola virus vaccine by the public.

**Methods:**

The study was a community-based cross-sectional qualitative and quantitative interventional study conducted in two communities, each in two states in Nigeria. An interviewer-administered questionnaire was used to collect information on respondents’ knowledge of the Ebola virus, the ways to prevent the disease, and their preventive practices, as well as their acceptability of and willingness-to-pay for a hypothetical vaccine against Ebola virus disease. The association between acceptability of the vaccine and other independent variables were evaluated using multivariate regression analysis.

**Results:**

Ebola virus disease was considered to be a very serious disease by 38.5% of the 582 respondents (224/582), prior to receiving health education on Ebola virus and its vaccine. Eighty percent (80%) accepted to be vaccinated with Ebola vaccine. However, among those that accepted to be vaccinated, most would only accept after observing the outcome on others who have received the vaccine. More than 87.5% was willing to pay for the vaccine, although 55.2% was of the opinion that the vaccine should be provided free of charge.

**Conclusion:**

The level of acceptability of Ebola virus vaccine among respondents was impressive (though conditional), as well as their willingness to pay for it if the vaccine is not publicly funded. In order to achieve a high uptake of the vaccine, information and education on the vaccine should be extensively shared with the public prior to the introduction of the vaccine, and the vaccine should be provided free of charge by government.

## Author Summary

Ebola virus disease (EVD) is highly virulent and transmissible. The transmission is mostly by direct contact with an infected person or indirectly through contact with material contaminated with the secretions or body fluids of an infected person. Currently there is no vaccine or drug for EVD. Maintaining good personal and environmental hygiene remains the only control strategy, and its implementation was a challenge in West Africa countries. Ebola virus vaccine (EVV) is being developed and may soon be deployed; thus a need to evaluate factors that can improve or discourage the uptake of the vaccine when it becomes approved for public administration. This study highlights the acceptability and willingness-to-pay for EVV. Majority of the respondents were willing to accept the vaccine and pay for it if it is not publicly funded. Of interest was that among those that accepted to be vaccinated, most would only accept to do so after they had observed the outcome on others that had received the vaccine. There is need for early dissemination of correct information and education on EVV to the populace so as to prevent any misinformation and misperception about the vaccine. This will improve universal coverage with the vaccine when deployed.

## Introduction

Ebola virus disease (EVD) is caused by Ebola virus (EBV), a highly virulent and infectious virus that infects humans and non-human primates. EVD is transmitted through human-to-human contact [1,2] and has up to 70% case fatality rate [3]. The current outbreak of EVD in six West African countries; Sierra Leone, Liberia, Guinea, Senegal, Mali, Nigeria [4] and reported cases in developed countries [5] have infected about 20,416 persons and caused 8,483 deaths [6,7] as at January 13, 2015.

The only available control strategy is strict personal and environmental hygiene, since no drug [8] has been approved for the treatment and no vaccine has been approved for its prophylaxis. The implementation of adequate hygiene; (avoiding contact with body fluids from an infected person or contact with items handled by an Ebola-infected patient, regular hand washing with soap and water and use of sanitizer) in West Africa is a challenge [9,10] principally due to poverty with existing low standard of living; lack of access to clean water, inadequate sanitation and overcrowded housing. Also inadequate health system in these countries lead to lack of or delayed case identification, inadequate supportive case management, contact tracing and surveillance which aid the spread of the disease. In view of the above limitations, effective prophylaxis through the introduction of Ebola virus vaccine (EVV) is urgently needed.

On August 28 2014, the National Institute of Health (NIH), USA announced that the first testing of EVV on humans by the National Institute of Allergy and Infectious Disease (NIAID) and GlaxoSmithKline (GSK) was imminent. The EVV, is a viral-vector-based recombinant vaccine in which genes encoding protein of Ebola virus is inserted into the genome of another virus (not Ebola virus), recombinant replication-deficient Chimpanzee-derived adenovirus 3 or cAd 3) [11] which when injected will generate both cellular and humoral immunity in the recipients. If approved for usage, the countries that have reported EBV cases may be among the first to benefit.

Whenever a new vaccine is introduced, it has to contend with public acceptability. Although, previous studies have reported favorable attitudes towards newly introduced vaccines [12–14] it would be an over assumption to conclude that introduction of the EVV will be welcomed with the same attitude and uncritical acceptance [15]. The existing poor knowledge on the vaccine by the uninformed masses [12], and the misconception of the possible risk of contracting an illness through a vaccine: as was attributed to oral polio vaccine [16,17], may dissuade majority from accepting the EVV with resultant low uptake of the vaccine, through propaganda [18,19].

Therefore, health program managers have to be proactive in identify early factors that can either facilitate or militate against the effective implementation of EVV program. Issues such as: the acceptance of the vaccine, making the decision to be vaccinated [20,21] and the willingness-to-pay (WTP) for EVV if not publicly funded need to be sorted out. The issue of willingness-to-pay for a newly introduced vaccine is paramount in Nigeria, since some vaccines that have previously been approved for public use are yet to be introduced in the National Programme on Immunization which is funded by the government. Therefore the aim of this study in Nigeria (West Africa) is to determine the public acceptability and willingness-to-pay for EVV. The outcome of this study will contribute to the strategic plan for a successful EVV implementation.

## Methods

### Study area

**Fig 1:**
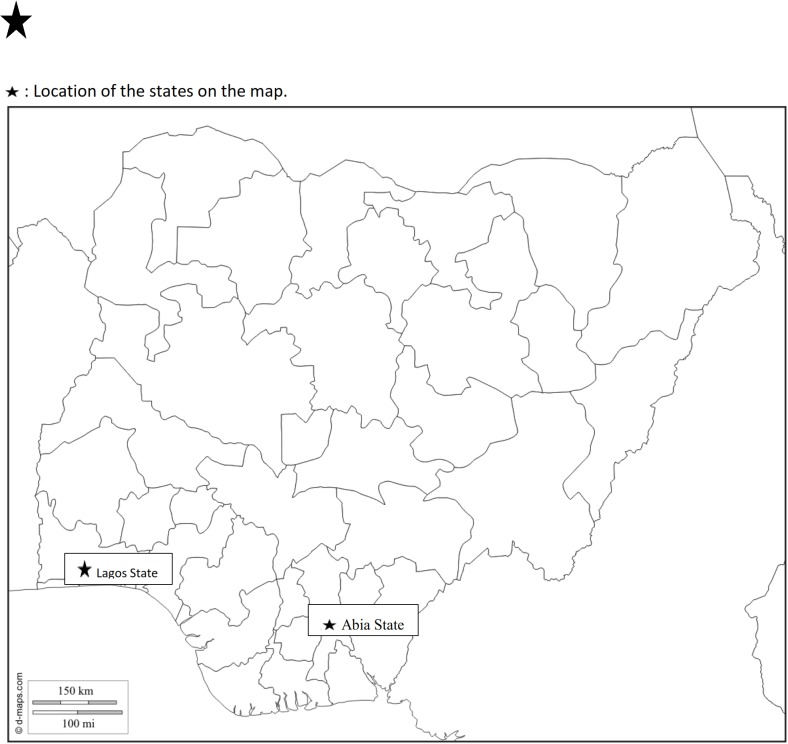
Map of Nigeria showing the study sites.

The study was conducted in two sites: an EBV low risk community (Umuahia), Abia State, Southeast and EBV high-risk community (Ajah) in Lagos State, Southwest, of Nigeria. The study took place from August to September 2014 during the period of Ebola outbreak in Nigeria, and data collection was completed before the Monday 20^th^ October 2014 when the World Health Organization (WHO) certified Nigeria free of EBV. The distance between the two communities is about 600 kilometers [22] (Fig 1). The first case of EVD in Nigeria was reported in Lagos in Ikoyi-Obalende Local Council Development Area (LCDA) about 22 kilometers from Ilaje community in Eti-Osa East LCDA, both administrative areas within Eti-Osa Local Government Area (LGA). By the time the WHO certified Nigeria free of EBV a total of 20 cases were reported out of which 8 deaths occurred. Among the reported cases and deaths, 19 cases occurred in Lagos State, out of which 7 deaths were recorded. Throughout the period, there was no report of EVD in Abia State.

Umuahia-North and Eti-Osa LGAs have populations of 359,230 [23] and 983,515 [24] respectively. The population density of Umuahia and Eti-Osa LGAs were 450 persons/km^2^ and 20,000 persons /km^2^ respectively. Ugba ward is one of the 12 wards in Umuahia-North and Ilaje is a community within Ward A of Eti-Osa East LCDA, one of the four council’s areas controlled by Eti-Osa LGA. The two communities are mixtures of both urban and rural areas.

### Study design

It was a community-based cross-sectional qualitative and quantitative interventional study conducted in two communities, each in two states in Nigeria. A stratified random sampling was used to select Ugba and Ilaje wards from a sample frame of 12 and 20 wards from Umuahia-North and Eti-Osa LGA respectively. A systematic random sampling was used to select the households from the house numbering done by the National Primary Health Care Development Agency. Households were selected, beginning with a house randomly selected and subsequent sampling was in alternate of four houses until the stipulated number was obtained. The minimum sample size of 260 for each study site was calculated using a power of 80%, 95% confidence level and based on the vaccine acceptability rate of 81.3% as reported by Williams et al [25]_._ The household heads participated in the study; if the head of the household was not around during the visit, the spouse was interviewed.

### Ethical clearance

The Health Research and Ethics Committee of the University of Nigeria Teaching Hospital (UNTH) Enugu, gave ethical approval for this study. The committee approved the use of only verbal consent from each respondent, the reason was to reduce contact between the researchers and their multiple respondents which would occur through exchange of writing materials. This was precautionary due to the EBV threat during the period of the study. Although a prior information sheet was given to the identified households seeking their consent to be part of the study, there was no signing of the counterpart consent sheet attached to the questionnaire.

### Data collection methods

The questionnaire was pre-tested in a community that was not involved in the final study. Few questions were modified to clear ambiguity and some translated words were changed to convey appropriate meaning. Also provisions were made to record comments made by the respondents, since it was realized that there were a lot of valuable information that were not originally included in the initial questionnaire design. The pre-tested interviewer-administered questionnaire was used to collect information on socio-demographic characteristics, respondent’s knowledge on EVD, preventive practices, attitude towards EVV, their knowledge and acceptability of EVV, and their WTP for EVV from the head of the family (S1 Text; sample of the questionnaire). Also information on the medium through which they first heard about Ebola virus disease outbreak was collected. One respondent per household, was interviewed. The preferred respondent was the head of the family, and in a situation where the father/husband was not available, his spouse was interviewed. If neither the head of the house nor the spouse was available to be interviewed, a second visit was rescheduled. The respondents’ acceptability and WTP were assessed pre and post health education on Ebola virus and its potential vaccine. They were informed that EVD is caused by Ebola virus (EBV) which is highly infectious and is transmitted through human-to-human contact [1,2]. That the virus has a very short incubation period and a victim manifests the disease within a very short time of exposure to the virus. The illness has 70% case fatality rate [3] and for a person to be protected by the vaccine, he/she has to receive the vaccine before exposure or not later than five days from the time of exposure. The EVV will be neither an inactivated vaccine which has been found to be unsuccessful with Ebola virus, nor live-attenuated vaccine which is generally considered too dangerous in the case of Ebola virus. The EVV will be a viral-vector-based recombinant vaccine in which genes encoding protein of Ebola virus will be inserted into the genome of another virus (not Ebola virus), a recombinant replication-deficient adenovirus (Ads) or attenuated vesicular stomatitis viruses (VSVs), which are known to cause no serious side effects or disease in human [26] The Ebola virus genes encoded proteins are recognized by the immune system and stimulate immune response against the disease but do not cause Ebola virus disease.

### Socioeconomic status (SES)

The inequality in WTP for EVV was done using the SES of the households [27,28] which was generated based on functional household asset they owned [29,30]. The household expenditure consumables were used to estimate the household income. The Principal Component Analysis (PCA) was used to create a continuous SES quartiles based on household asset owned and expenditure on food [31]. It is easier to elicit monetary information, like income [32,33] with this approach. The estimation was in Nigerian naira (N) and converted at the rate of 1 United States dollars (USD) to N170.00.

### Respondents’ history on EVD

Respondents were asked to recall the first medium through which they got to know about EVD, and multiple options were not allowed. This was to determine the effective channel of disseminating information to the wider population. They were also asked to state their perception of the severity of the disease when they first heard about of EVD and this was to indirectly assess the content and impact of the first information on the public awareness on the disease.

### Knowledge on how EVD can be prevented and their preventive practices

Respondents were asked questions to elicit their knowledge on ways EBV could be contracted, ways to prevent Ebola virus infection, and their preventive practices. They were also tested on their awareness of any vaccine against EVD. The respondents were allowed to give their reply to the questions without pre-empting them with answer options. There were five possible correct preventive measures, as well as preventive practices [34]. A normative value of 1 or 0 was given for correct and wrong responses respectively for the questions. For their knowledge on how EBV could be prevented, a score of 1 was given for any correct response. The lowest and highest possible scores for preventive knowledge were 0 and 5 respectively. The respective total scores were grouped into “very adequate” if 5, “adequate” if 3–4 and “inadequate” if 0–2. Adequacy means that the respondent scored at least three on knowledge of prevention of Ebola virus infection.

On the evaluation of practice, personal hygiene which has two major components was assessed. Nigeria had only 20 cases of EBV and 8 deaths. Therefore most of the respondents had neither seen a person suffering from EVD nor seen someone die from it.

### Acceptability of EVV

The respondents were asked whether they knew of any EVV. Those that responded stated that: a) there was a vaccine but not approved for use or any related response, were categorized as “correct” and those that stated that: b) there was a vaccine available to combat EBV, or any related response were categorized as “wrong”. To assess the respondents’ acceptability of EVV, a hypothetical cAd3 [17] was described to them. They were informed that the vaccine would be safe and may cause little or no adverse events and lacks the potential of causing the disease. Their willingness to be vaccinated was elicited. Those that accepted to be vaccinated were asked their preferred time to receive the vaccine. A 5-point Likert scale scoring system ranging from 1 = “very unwilling”, 2 = “unwilling”, 3 = “not sure’, 4 = “willing” and 5 = “very willing” was used to rate their level of acceptability of EVV. The respondents who replied either “1’,”2” or “3” were categorized as unwilling, while those whose responses were either “4” or “5” were grouped as willing.

### WTP for EVV

The WTP for the hypothetical EVV was evaluated only among those who accepted to be vaccinated. Their WTP for the vaccine was determined using the contingent valuation method. The highest amount that they were willing to pay for the vaccine was sought after. Since the vaccine is yet to be deployed to the market, no market price is yet available. The contingent valuation method (CVM), is a survey-based approach to elicit monetary valuation of products of healthcare [35,36] by individuals’ using bidding game approach (BGM). This is best suited for exploring individual preferences for goods and services with no known market price, as in this case where there is no market price for EVV. The respondents were presented with a scenario describing the hypothetical EVV, as an effective injectable vaccine, with no risk of getting infected with Ebola virus through the vaccine and should be given preferably before exposure to Ebola virus or at most within five days of exposure. Since no market price was available for the vaccine, the respondents were asked an open ended question: “*How much will you be willing to pay for the Ebola virus vaccine?*” After the initial response, they were allowed one more option to either increase or reduce the initially stated amount by a second question: “*If due to inflation or other uncertainties*, *the cost for the vaccine is higher than what you have just stated*, *what is the maximum amount you are very certain to pay bearing in mind that your entire household (both adults and children) may have to receive the vaccine at about the same period?*” The effect of cost of vaccine on acceptance rate was elicited by comparing the willingness to vaccinate before knowledge on cost and thereafter. Any other important comments made were documented and analyzed as direct speech.

### Data analysis

Certain variables were dichotomized into binary variables: educational status into primary education or less and secondary education and above, acceptability into before and after knowledge on WTP. The households were categorized into those with household size of 1–3 and those of 4 and above. The two major components of practice care hygiene, were each scored 0.5 and a total score of 1 = adequacy and 0.5 = inadequacy. Association of other variables with acceptances of EVV was tested using univariate and multivariate analysis. Two-by-two table was created to test for statistical significance and p-value of <0.05 was taken to be statistically significant. The continuous socio-economic status (SES) index was generated using Principal components analysis (PCA) based on combination of household assets owned and mean weekly expenditure on food items. The SES index was categorized into four equal quartiles (Q) and these four groups were: the poorest (Q1), very poor (Q2), poor (Q3), and least poor (Q4). The correlation between SES and WTP was measured using SES groups.

## Results

**Fig 2:**
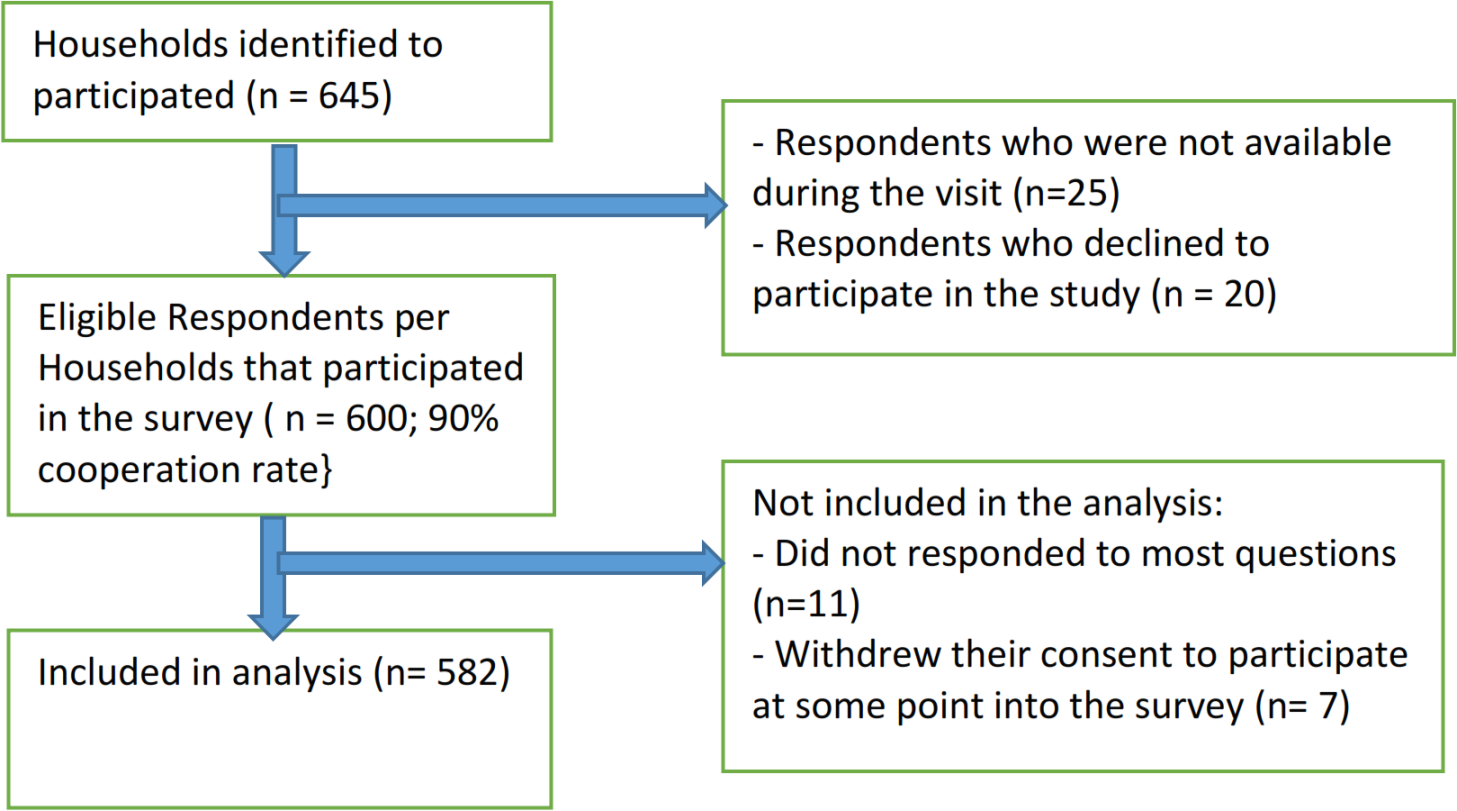
Sample selection framework.

A total of 645 households were identified for the study and 25 households had neither the father nor the mother to be interviewed while 20 households, the identified respondents declined to participate in the study. Therefore, 600 households were studied giving a cooperation rate of 93%. Of the 600 respondents that participated, 18 questionnaires were not analyzed, the reason being that 11 respondents did not give any responses to most of the questions in the questionnaire and 7 withdrew their consent to participate at some point during the interview (Fig 2).

The Ilaje community had more male respondents (73.3%, 215/293) while (54.1%, 133/289) of the respondents in Ugba were females. The mean ages of the respondents were 37.2 years and 38.32 years in Ilaje and Ugba respectively. In Ilaje and Ugba communities 93.3% (271/293) and 86.2% (249/289) respectively have at least secondary education. The occupation of most of the respondents; 34% (100/293) and 35.6% (103/289) in Ilaje and Ugba, respectively, was small scale business (see S1 Table).

### Source of EVD knowledge

**Fig 3:**
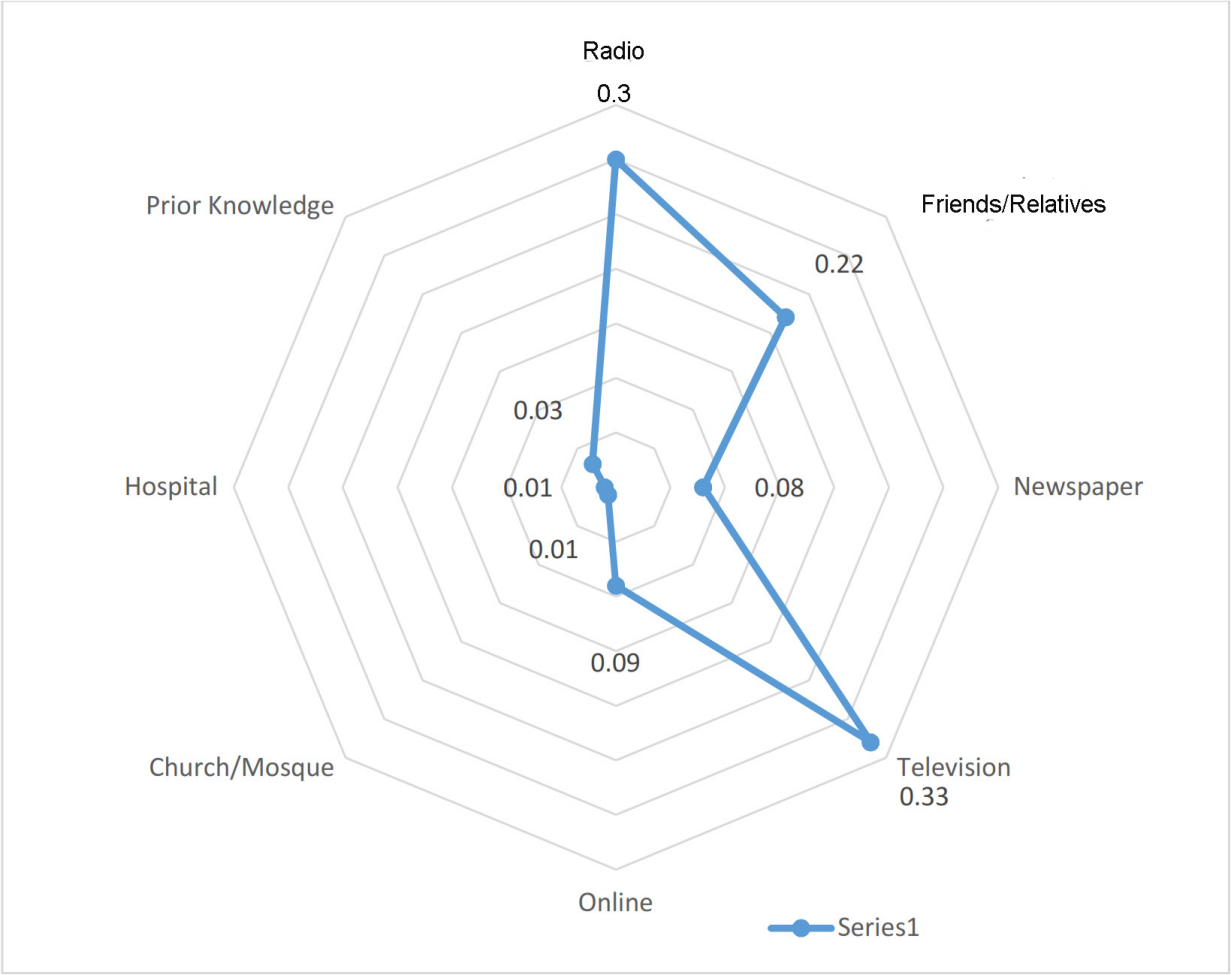
Respondents’ source of knowledge on EBV.

The media through which most of the respondents first learnt about EVD were television (32.4%, 178/582) and radio (27.1%, 150/582). No one heard it from either church/mosque (0.0%) or hospital (0.0%) (Fig 3).

Ninety five percent of respondents stated that EBV can be transmitted by contact, Table 1. Majority (73%, 425/582) mentioned washing of hands as a preventive measure. Only 38.5% (224/582) appreciated the seriousness of the disease when they heard of the EVD for the first time. Hand washing (66.7%, 388/582) was the commonly adopted preventive measure while 12.3% (72/582) took no precautions.

**Table 1:** Responses to questions assessing knowledge and EBV preventive practices since the EBV epidemic threat.

Questions	Correct Knowledge Responses (%)	Correct EBV Prevention Practice (%)
EBV can be contracted through:		
i) Contagious/contact	552 (94.9)	
ii) Food borne	30 (5.1)	
Can EBV infect anybody?	418 (71.8)	
EBV Infection can be prevented through:		
i) Wash hand frequently	425 (73.0)	388 (66.7)
ii) Avoid contacts (handshaking/hugging)	221 (43.2)	134 (23.1)
ii) Keep clean environment	220 (37.8)	131 (20.5)
iv) Use germ killer (sanitizers) frequently	141 (24.3)	136 (22.6)
v) Avoid bats/monkeys	63 (10.8)	0 (0.00)
vi) Avoid contaminated items with infective body fluids	16 (2.7)	0 (0.00)
vii) Avoid corpse of an EBV patient/contaminated hospital	0.0	0 (0.00)
viii) ***Prayer/God’s protection***	224 (38.5)	0 (0.00)
ix) ***Takes no precaution***	72 (12.3)	0 (0.00)
The Mean [SD] of the total correct answers.	1.92 [0.77]	0.81
Inadequate	492 (84.6)	145 (25.0)
Adequate	90 (15.4)	436 (75.0)
Very adequate	0 (0.0)	0 (0.00)
EVD considered a serious illness *ab-initio*.	224 (38.5)	

### Acceptance of EVV

The respondents in Ilaje and Ugba that acknowledged that there was an EVV were 2.7% (8/293) and 41.0% (119/289) respectively (p = 0.0001) (Table 2). Prior to health information on EVV, 80.0% (234/293) and 79.5% (230/289) of the respondents in Ilaje and Ugba respectively would accept EVV (p = 0.93). After information on EVV, 86.3% (253/293) (Ilaje) and 82.1% (237/289) (Ugba) were willing to be vaccinated. Among those that accepted to vaccinate once EVV is available in Ilaje and Ugba were 79.8% (202/253) and 47.7% (113/237) respectively, while 12.2% (31/253) and 51.5% (122/237) in Ilaje and Ugba would like to receive the vaccine later, after observing the effect on those that received it (p = 0.0001). The respondents that were unwilling to vaccinate in Ilaje and Ugba were 6.9% (16/233) and 37.8% (90/237) respectively (p = 0.0001). Among those willing to vaccinate, 91.2% (212/233) and 87.5% (207/237) in Ilaje and Ugba respectively were willing to pay for EVV (p = 0.2). The very poor and the least poor were willing to pay the least amount of money for the vaccine, while the poorest and the poor were willing to pay a higher amount for the EVV. Households with household size of 4–5 in number were willing to pay the highest amount of money for the EVV (S2 Table).

**Table 2:** Acceptance and WTP for EVV.

Questions	Ilaje (Lagos) n = 293	Ugba (Abia) n = 289	*Χ* ^2^	*p-value*
Is there EVV?				
Yes	8 (2.7)	119 (41.0)		
No	273(93.1)	59 (20.5)	126.06	0.0001
Don’t know	12 (4.2)	111 (38.5)		
Will you vaccinate EVV if available (pre-information)?				
Yes	234 (80.0)	230 (79.5)		
No	25 (8.5)	52 (18.1)	0.01	0.93
Don’t Know	34 (11.5)	7 (2.4)		
Post information acceptability of EVV? (n^1^ = 293, n^2^ = 289)				
Yes	253 (86.3)	237 (82.1)	2.06	0.1511
No	40 (13.7)	52 (17.9)		
When will you prefer to vaccinate? (n^1^ = 253, n^2^ = 237)				
i) Immediately	202 (79.8)	113 (47.7)		
ii) Later, after observing outcome on others	31 (12.2)	122 (51.5)	53.7	0.0001^β^
iii) Don’t know	20 (8.0)	2 (0.8)		
Level of acceptance of EVV? (n^1^ = 233, n^2^ = 237)				
Mean willingness (Max. is 5).	4.3	3.7		
Unwilling	16 (6.9)	90 (37.8)	65.09	0.0001
Willing	217 (93.1)	147 (62.2)		
WTP for EVV? (n^1^ = 233, n^2^ = 237)				
Yes	212 (91.2)	207 (87.5)		
No	21 (8.8)	30 (12.5)	1.61	0.2
Amount WTP (n^1^ = 212, n^2^ = 207)				
Average Amount USD (naira)	10.8(1833.9)	15.3(2602.0)		
≤USD5.9 (≤1000naira)	0.0	91 (43.9)		
>USD5.9 - ≤ 29.4 (>1000 - ≤ 5000naira)	203 (95.7)	94 (45.2)		
>USD29.4 (>5000naira)	9 (4.3)	22 (10.9)	N/A	N/A

n^1^ & n^2^ = analyzed sample size in Ajah & Umuahia respectively

^β^ = Yates corrected.

Some of the respondents who were not willing to pay for the EVV as well as some of those who were willing were of the opinion that government should provide the vaccine at no cost to the recipients (commented by 55.2%). Respondents stated that “*Government should pay for it and make it free*.” *“It is among the duties of the government to protect the citizens and providing this vaccine should be one way to do that”*. *“I don’t think it is right to expect people to pay for a vaccine that will protect them from a disease they do not have any control on how it can be contracted*.*”* Others suggested that government should coerce people to receive the vaccine. “*EBV disease is a public health problem*” and “*Government should persuade everybody to receive the vaccine*, *and the only way they can do that is to provide it free of charge”*. *“It should be made compulsory and enforced*. *If it would be enforced*, *you cannot ask people to pay for it*.*”* Other common suggestions on how to avoid or minimize out-of pocket payment by the people (reported by 21.0%) were: *“National Health Insurance Scheme (NHIS) should cover the cost*.*” “The vaccine bill should be incorporated into the costs of GSM phone bills…*.*”*

### Determinants that may influence acceptance of EVV

Univariate and multivariate analyses demonstrated that educational status strongly correlated with acceptance of EVV (Table 3). The lower the educational status, the more likely they are to accept the vaccine (95% CI: 0.20–0.74, p-value = 0.001). It was also shown that giving health education on EVV improved its acceptability and the difference found to be statistically significant (95% CI: 0.54–1.01, p-value = 0.046)

**Table 3:** Determinants among respondents that may influence their acceptance of EVV univariate and multivariate analysis.

Variables	Vaccine Acceptance Favorable	Vaccine Acceptance Unfavorable	p-value	Crude OR	95% CI	Adjusted OR	95%CI	p value
District								
Ilaje	253	40	2.06	1.39	0.87–2.23			
Ugba	237	52						
Religion^1^(n = 582)								
Christian	399	91	0.001 ^β^	0.04	0.0–0.32	0.82	0.79–0.86	0.002
Muslim	91	1						
Gender								
Male	293	55	0.998	1.00	0.62–1.61			
Female	197	37						
Household size (n = 581)								
≤ 5	352	60	0.25	1.32	0.80–2.18			
≥ 6	138	31						
Educational status^1^(581)								
No-formal/Primary	42	18	0.001	0.39	0.20–0.74	0.81	0.69–0.96	0.002
& Secondary/Tertiary	448	73						
Occupation (n = 576)								
Unemployed	40	9	0.58	0.81	0.36–1.86			
Employed	182	31						
Self-employed	264	50						
Ebola Health Education‡								
Pre-Health Education	464	118	0.046	0.74	0.54–1.01	0.93	0.75–1.10	0.05
Post-Health education	490	92						
Socioeconomic quartile (n = 580)								
Q1/Q2	237	52	0.30	0.79	0.50–1.26			
Q3/Q4	248	43						

^1^ included in multivariate model.

^β^ Yates corrected.

‡ Summation of favorable and unfavorable responses from the study sites pre and post health education.

## Discussion

The study showed that majority of the respondents learnt about EVD from the local media (television and radio) and none heard it from health care worker. This is similar to what have been reported elsewhere [37,38]. This could be partially attributed to the prevailing industrial action which closed the services in many public medical facilities during the early period of EBV threat in Nigeria. Although, private sector healthcare providers were functional, it has been revealed that healthcare providers in developing countries rarely educate their clients/patients on health related issues [39]. The lack of healthcare providers role in providing initial information on EVD, may account for the high proportion of the respondents that did not understand and appreciate the seriousness of the disease. Healthcare providers should assist the public at every contact in obtaining health information to optimize their health outcome.

Majority of respondents identified personal hygiene as an effective preventive measure, but practiced avoidance of casual hand shake as a preventive action, thus, revealing a gap between knowledge and practice. It has been established that EBV can spread from person to person through contact with body fluids from an infected individuals’ blood, feces, or other body fluids, not by avoidance of handshakes, hugging or being in a gathering like most respondents stated [40].

The level of acceptability of EVV among all socioeconomic classes was high. This high acceptability may not translate to prompt EVV uptake since majority would like to observe the effect of the vaccine on others before vaccination. The delay by some people in receiving the vaccine may segregate the population and provide an opportunity to spread inaccurate vaccine information which may lead to stigmatization of those that have received the vaccine [41,42] Healthcare providers should strive to gain public trust on the new vaccine by providing information on safety and side effects [43]. Respondents’ comments such as “*EBV disease is a public health problem and Government should compel everybody to receive the vaccine…*.*”* and *“It should be made compulsory and enforced*.*”* are calls for strong political will to ensure public acceptance of EVV. Government should play a pivotal role in sharing the correct information about EVV with the people, Also as stated by one of the respondents, “*It is among the duties of the government to protect the citizens and providing this vaccine should be one way of doing that…*.,”. However the government should not stop at providing this vaccine free of charge to the community, but encourage and possibly, coerce people to receive it. The classification of the current EVD outbreak as a ***public health emergency of international concern*,** justifies the use of reasonable legal measure to control the outbreak, including the option of compelling people to get vaccinated.

Majority of the respondents were WTP for EVV. This is similar to what has been reported on other new vaccines [44]. This may be due to the anticipated benefit which influences decision to pay for a product [45] In this case, it is the protection from EBV, since the risk of infection was high during the outbreak. However, no information was available on the market price to compare the average amount they were willing to pay for EVV. Most new drugs and vaccines are not affordable for out-of-pocket payment especially for the poorer households. Therefore, government should make plans to either subsidize the cost of the vaccine or at best bear the full cost. The suggestion by some respondents for NHIS to cover the cost of EVV may not be an ideal option, in a country like Nigeria, where NHIS currently covers only employees in the Federal formal sector which accounts for less than 5% of the population [46]. In Nigeria, WTP studies have been conducted for several healthcare products and services [47–49]. However, we are not aware of any WTP study in both Nigeria and beyond with respect to pre-vaccine deployment for EVV. Thus there is little information on how this can affect the accessibility of the vaccine when introduced. Nonetheless, the envisaged assistance of Global Alliance for Vaccine Initiatives (GAVI) and UNICEF with funds for the vaccine would increase the possibility of no financial cost to recipients when EVV is deployed in many countries that reported outbreak of EVD. However, a major obstacle that these nations will encounter is establishing a long-term financial sustainability structure that will continue to maintain accessibility and affordability of EVV if in future there is donor fatigue and reduction in funds. This raises the pertinent issue on how much people will be willing to pay for EVV. Furthermore, government may in an effort to make the EVV easily accessible to the public in situation of lack of external sponsor, find it necessary to subsidize the cost of the vaccine. In such a situation, government may need information on willingness-to-pay for the vaccine.

One of the limitation of this study is that it was conducted during the outbreak of EBV disease in the West African sub-region including Nigeria. The reality and fear of potential EBV infection might have affected the responses obtained. The findings of our study could be a representative of the highest level of acceptability and WTP for EVV. A concurrent study in any of the western countries where the threat of EBV was virtually non-existent might reveal a lower acceptability. Secondly, the hypothetical nature of the study may differ from the real practice. However the findings give an insight to the possible challenges that may exist when the real vaccine is introduced. Another limitation is the use of ownership of household assets in the classification of SES, in community where people purchase either used cheap items or brand new expensive items. This has the potential of leading to wrong SES classification. The most objective assessment would have been classification based on income, but previous experiences have shown that obtaining such information is often shrouded with difficulty. The other socioeconomic classification approach by Oyedeji or Olusanya which relies on highest education and occupation of the parents was not used.

### Conclusion

The level of acceptability of Ebola virus vaccine among respondents was high, although majority confirmed that they would not hastily receive the vaccine until they observe the effect on others. Nonetheless, they was willingness to pay for the vaccine whenever they are to receive it should the vaccine not be publicly funded. However, it is recommended that for high uptake to be achieved, the vaccine introduction should be preceded with wide public health education, counselling and persuasion, and government should endeavor to provide the vaccine free of charge.

## Supporting Information

S1 Checklist STROBE checklist. (DOC)

S1 Text Questionnaire used in the study. (DOCX)

S1 Table Characteristics of the respondents in the two localities. (DOCX)

S2 Table Discrepancies in WTP for EVV according to different socioeconomic quartiles and household size. (DOCX)
