# Supplementary material for: Acceptability and Willingness-to-Pay for a Hypothetical Ebola Virus Vaccine in Nigeria
Source: PLoS Negl Trop Dis. 2015 Jun 15;9(6):e0003838. doi: 10.1371/journal.pntd.0003838 (PMC4467844; doi:10.1371/journal.pntd.0003838)
Supplement: S1 Checklist — (DOC) [file pntd.0003838.s001.doc]

STROBE Statement—Checklist of items that should be included in reports of ***cross-sectional studies***

|  | Item No | Recommendation |
| --- | --- | --- |
| **Title and abstract** | 1 | (*a*) Indicate the study’s design with a commonly used term in the title or the abstract  To avoid lengthy title the study design was included in the abstract and the body of the manuscript. |
|  |  | (*b*) Provide in the abstract an informative and balanced summary of what was done and what was found  These have been included. |
| Introduction | | |
| Background/rationale | 2 | Explain the scientific background and rationale for the investigation being reported  The background was written to reflect the rationale for the study. |
| Objectives | 3 | State specific objectives, including any pre specified hypotheses.  The aims of the study were included. |
| Methods | | |
| Study design | 4 | Present key elements of study design early in the paper.  The study design was structured such that the important elements of the design were in the early part of the paper. |
| Setting | 5 | Describe the setting, locations, and relevant dates, including periods of recruitment, exposure, follow-up, and data collection  All these are included in the methods. |
| Participants | 6 | (*a*) Give the eligibility criteria, and the sources and methods of selection of participants.  These have been included. |
| Variables | 7 | Clearly define all outcomes, exposures, predictors, potential confounders, and effect modifiers. Give diagnostic criteria, if applicable  Not applicable in this study. |
| Data sources/ measurement | 8* | For each variable of interest, give sources of data and details of methods of assessment (measurement). Describe comparability of assessment methods if there is more than one group.  Not applicable in this study. |
| Bias | 9 | Describe any efforts to address potential sources of bias.  The systematic method used in the selection of respondents removed bias. |
| Study size | 10 | Explain how the study size was arrived at.  This is included in under study design. |
| Quantitative variables | 11 | Explain how quantitative variables were handled in the analyses. If applicable, describe which groupings were chosen and why.  This is explained under data analysis, as well as under each variable tested. |
| Statistical methods | 12 | (*a*) Describe all statistical methods, including those used to control for confounding  The statistics used were described. |
|  |  | (*b*) Describe any methods used to examine subgroups and interactions |
|  |  | (*c*) Explain how missing data were addressed  This was explained in the result section. |
|  |  | (*d*) If applicable, describe analytical methods taking account of sampling strategy  Not applicable in this study. |
|  |  | (*e*) Describe any sensitivity analyses  Not applicable in this study. |
| Results | | |
| Participants | 13* | (a) Report numbers of individuals at each stage of study—eg numbers potentially eligible, examined for eligibility, confirmed eligible, included in the study, completing follow-up, and analysed  This has been included in the first paragraph of the result section. |
|  |  | (b) Give reasons for non-participation at each stage  The reasons for none participant |
|  |  | (c) Consider use of a flow diagram  These have been included. |
| Descriptive data | 14* | (a) Give characteristics of study participants (eg demographic, clinical, social) and information on exposures and potential confounders  Described in the supplementary table. |
|  |  | (b) Indicate number of participants with missing data for each variable of interest |
| Outcome data | 15* | Report numbers of outcome events or summary measures |
| Main results | 16 | 1. Give unadjusted estimates and, if applicable, confounder-adjusted estimates and their precision (eg, 95% confidence interval). Make clear which confounders were adjusted for and why they were included   Not applicable in this study. |
|  |  | (*b*) Report category boundaries when continuous variables were categorized  This was described in the method section. |
|  |  | (*c*) If relevant, consider translating estimates of relative risk into absolute risk for a meaningful time period |
| Other analyses | 17 | Report other analyses done—eg analyses of subgroups and interactions, and sensitivity analyses |
| Discussion | | |
| Key results | 18 | Summarise key results with reference to study objectives  The discussion was structured to follow the key findings. |
| Limitations | 19 | Discuss limitations of the study, taking into account sources of potential bias or imprecision. Discuss both direction and magnitude of any potential bias  Limitations have been stated and explained. |
| Interpretation | 20 | Give a cautious overall interpretation of results considering objectives, limitations, multiplicity of analyses, results from similar studies, and other relevant evidence |
| Generalisability | 21 | Discuss the generalisability (external validity) of the study results  Stated in the conclusion |
| Other information | | |
| Funding | 22 | Give the source of funding and the role of the funders for the present study and, if applicable, for the original study on which the present article is based  There was no funding for the study. |

*Give information separately for exposed and unexposed groups.

**Note:** An Explanation and Elaboration article discusses each checklist item and gives methodological background and published examples of transparent reporting. The STROBE checklist is best used in conjunction with this article (freely available on the Web sites of PLoS Medicine at http://www.plosmedicine.org/, Annals of Internal Medicine at http://www.annals.org/, and Epidemiology at http://www.epidem.com/). Information on the STROBE Initiative is available at www.strobe-statement.org.
