# Supplementary material for: Acceptability and Willingness-to-Pay for a Hypothetical Ebola Virus Vaccine in Nigeria
Source: PLoS Negl Trop Dis. 2015 Jun 15;9(6):e0003838. doi: 10.1371/journal.pntd.0003838 (PMC4467844; doi:10.1371/journal.pntd.0003838)
Supplement: S1 Text — (DOCX) [file pntd.0003838.s002.docx]

**Impact of awareness and disease scare on willingness to receive Ebolavirus vaccine**

**QUESTIONNAIRE**

Dear respondent,

We are interested in determining people’s attitude towards Ebola virus disease. Ebola is a highly contagious disease that can cause internaland external bleeding and frequently cause death. Presently there is no confirmed cure for it. Prevention is the only measure to combat the disease. This is achieved principally through universal precautions of frequent washing of hands by everybody and, barrier measures by healthcare providers.. Currently a lot of efforts are being made to develop vaccine that can prevent the disease. Hence, we want to interview you regarding you perception of the disease and whether you will be willing to accept preventive interventions. The outcome of this survey will be helpful in policy decision. Your answers to these questions will be kept confidential and your participation is voluntary. You do not have to answer any question you do not want to answer. You are free to ask any question now.

**Pre – interview Information**.

Code number:………

Respondent’s home address (street and location)………………………………… Contact phone number (optional)…………………….…

**SECTION 1. DEMOGRAPHIC AND SOCIO-ECONOMIC INFORMATION**

1. How old are you? ......................... 2. Respondent’s gender ……………………………

3. Ethnicity? Ibo [ ], Yoruba [ ], Hausa/Fulani [ ] others [ ] Specify……………………………………

4. Religion? Christianity [ ], Islam [ ], Traditional [ ], Others [ ] specify …………………………………..

5. Marital status? Single [ ], Married [ ], Divorced [ ], Widowed [ ]

6. What is the size of your household? Adult [ ] + Children {≤ 18 years} [ ] = [ ]

7. Do you consider yourself to be the main decision-maker on issues concerning your household health care? [ ] yes, [ ] No.

8. Did you go to school? [ ] yes [ ] No. ***If yes go to question*** 9***, if no go to question***10.

9. What was the highest completed education level? ***(Please use “√” for the respondent and mark “# “for the partner)***

Primary [ ] Junior Secondary [ ] Senior Secondary [ ] University [ ]

10. What occupation is your major source of income? ***(Please use “√” for the respondent and mark “# “for the partner)***

Farmer [ ]

Unemployed professional [ ]

Unemployed non-professional [ ]

Petty trading [ ]

Junior Civil Servant [ ]

Senior Civil servant [ ]

Employed in private sector [ ]

Self-employed Businessman [ ]

Others [ ] Please specify……………………………

**SECTION 2. HISTORY ON EBOLA VIRUS:**

11. Have you heard about Ebola virus? [ ] yes, [ ] No.

12. Confirm the medium through which you heard it first?

Radio [ ], Friend/Relative [ ], Newspaper [ ], Television [ ], Online (Internet/facebook/tweeter etc) [ ], Church [ ], Hospital [ ]Read it from textbook [ ], Can’t remember [ ] Any other medium [ ]

Please state………………………………………………………….

13. Did you appreciate the seriousness of the disease when you heard about it for the first time? [ ] yes [ ] No = 0.

14. What made you to realize the seriousness of the disease?

Someone died from it [ ], Heard it is easy to contract [ ], Heard no cure for it [ ] Others [ ] Specify..........................

15. What are the ways through which Ebola Virus is contracted?

Air borne [ ], Water borne [ ], Insect borne [ ] Contagious/contact [ ], Food borne [ ]

Any other [ ] Specify………………………….

16. Do you believe that anybody including you can contact Ebola virus? [ ] yes,[ ] no.

17. What measures do you think should be taken to prevent spread of Ebola virus? ***(Please do not pre-empt)***

(a) Wash hands frequently [ ] (b) Clean house and compound thoroughly [ ] (c) Do not shake hands or hug people [ ] (d) Use germ killer to wipe hand (sanitizers) frequently [ ] (e) Stop eating bush meat [ ]

(f) Protect from contact with bats, monkeys [ ] (g) Use native medicines [ ]

(h) Others Please specify………………………………

18. What measures have you been taking to prevent yourself from being infected with Ebola virus, since the onset of the disease threat?

(a) Wash hands frequently [ ] (b) Clean house and compound thoroughly [ ] (c) Do not shake hands or hug people [ ] (d) Use germ killer to wipe hand (sanitizers) frequently [ ] (e) Stop eating bush meat [ ]

(f) Protect from contact with bats, monkeys [ ] (g) Use native medicines [ ] (h) Others Specify………………………..

**ACCEPTABILITY OF EBOLA VIRUS VACCINE**

19. Is there any vaccine available to prevent Ebola virus disease? [ ] yes, [ ] No, [ ] don’t know.

20. If there is Ebola virus vaccine, will you accept to be vaccinated? [ ] yes, [ ] No, [ ] don’t know.

21. When will you prefer to receive the vaccine?

During this period of high prevalence and transmission [ ], Later, after this period of high transmission [ ]

Don’t Know [ ].

22. If you are not willing to be vaccinated, what is/are your reason(s) for your hesitancy towards Ebola virus vaccine?

(***Please probe for reason but don’t pre-empt)***

a) The media have not promoted the vaccine well enough [ ],

b) It is all political [ ]

c) My socio-cultural beliefs do not encourage vaccines [ ]

d) My religious beliefs do not promote vaccines [ ]

e) Our leaders have not informed us to do so [ ]

f) Vaccines have risks/adverse events [ ]

g) My experience with past vaccination is discouraging [ ]

h) Ebola can still infect anybody, both vaccinated and unvaccinated [ ]

i) It is an injection and will cause pain [ ]

j) Others should receive it first to let me see the effect before receiving myself [ ]

k) I am concerned about the competence of vaccinators

l) Ebola vaccine should not be a top priority now [ ]

m) There is rumour that you can get infected through the vaccine [ ]

n) Probable cost of the vaccine [ ]

o) Any other reasons [ ]…………………………………………………………………………………………….

**Ebola virus disease is caused by Ebola virus which is highly infectious and is transmitted through human-to-human contact. The virus has a very short incubation period and a victim manifests the disease within a very short time of exposure to the virus. It has high fatality rate, and for a person to be protected by the vaccine, he/she has to receive the vaccine before exposure or not later than five days from the time of exposure. There is a vaccine being developed to protect people from this killer disease. The process of developing the vaccine has ensured that the vaccine cannot cause the disease. The EVV will be neither an inactivated vaccine: which has been found to be unsuccessful with Ebola virus, nor live-attenuated vaccine: which are generally considered too dangerous in the case of Ebola virus. The EVV will be viral-vector-based recombinant vaccine in which genes encoding protein of Ebola virus will be inserted into the genome of another virus (not Ebola virus): recombinant replication-deficient adenovirus (Ads) or attenuated vesicular stomatitis viruses (VSVs), which are known to cause no serious side effects or disease in human. The Ebola virus genes encoded proteins are recognized by the immune system but do not cause Ebola virus disease. For the vaccine to be effective, it has to be received within 24-48 hours of contacting the virus, otherwise it will not be beneficial to the recipient. Alternatively, it can be received as a single or two-dose vaccine days before exposure to infected person, for the body to develop the much needed immunity.**

22. With this information, will you be willing to accept Ebola virus vaccine if it is offered in any immunization facility?

Yes [ ] No [ ]

23. Please rate your level of acceptance of Ebola vaccine?

Very unwilling [ ], Unwilling [ ], Not sure [ ], Willing [ ], Very willing [ ]

**WILLINGNESS TO PAY (WTP) FOR EBOLA VIRUS VACCINE**

**WTP FOR SELF**

24. If the Ebola vaccine is not publicly funded,will you be willing to pay for it? [ ] yes, [ ] No. ***if Yes go to 25, if No go27.***

25. How much will you be willing to pay for the Ebola virus vaccine? [ ] naira.

26. If due to inflation or other uncertainties, the cost for the vaccine is higher than what you have just stated, what is the maximum amount you are very certain to pay bearing in mind that your entire household (both adult and children) may have to receive the vaccine about the same period? [ ] naira.

**SECTION 4. WEEKLY FOOD COSTS AND OTHER EXPENDITURES:**

This is information to determine the socio-economic status of the family.

27. How much did your household spend to purchase food from the market in the **PAST ONE WEEK** on the various items that are listed below? Even, if the food items consumed were produced by your household produced, if bought from the market, how much will they cost?

| **Item** | **Quantity** | **Cost (Amount)** |
| --- | --- | --- |
| Gari/Wheat/Semovita etc |  |  |
| Beans |  |  |
| Cassava (akpu)/Fufu/Amala |  |  |
| Rice |  |  |
| Yam |  |  |
| Fish (Fresh, Dry, Ice) |  |  |
| Meat (Chicken, Beef, Goat, Snail etc) |  |  |
| Vegetables ( |  |  |
| Others (specify) |  |  |
| **Total** |  |  |

28. About how much did the household spend in the past month on the following items listed below?

a. Firewood [ ] b. Kerosene [ ] c. Charcoal/coal [ ]

d. Electricity [ ] e. Candles [ ] f. Cooking gas [ ]

g. Torch batteries [ ] h. Petrol/Diesel for generator [ ] i. Others (Specify)[ ]…………………

**Grand total [ ] naira.**

**SECTION 5. HOUSEHOLD ASSET HOLDINGS:**

29. Tell me whether your household owns these functional items?

**Electronic** {a. Radio [ ] b. Fridge [ ]c. Television [ ]} **Transport**d. {Bicycle [ ] e. Motorcycle [ ] f. Motorcar [ ]}

**Lighting {**g. Kerosene lamp ] h. Generator [ ] i. Rechargeable lamp [ ]}

CERTIFICATE OF CONSENT FOR THE STUDY ON**“ IMPACT OF AWARENESS AND DISEASE SCARE ON WILLINGNESS TO RECEIVE THE EBOLA VIRUS VACCINE”**

I (Researcher) would like to ask you some questions concerning yourself, and knowledge on Ebola virus, preventive measures you have been taking and whether you are willing to accept any other Ebola preventive measures if introduced. I shall not give you any money for participation in this study.However your participation will be helpful in policy reform on palliative care. I want to assure you that I shall keep everything I discuss with you in confidence. You are free not to answer any question you do not wish to answer. However I appreciate your contribution.

I (Respondent) was requested to participate in the study of Ebola virus disease and issues concerning it . I willingly volunteered to be part of the study, I have read the consent form / the information on the consent form has been read to me. I had opportunity to ask questions, and to answer the questions that were asked of me, I answered them sincerely as much as I can. I also know that I can withdraw from the study at any time, and my withdrawal will not affect my health.

Print Name of Respondent ……………………………….Date and Signature of Respondent………………………………….

Print Name of Researcher…………………………… Date and Signature of Researcher……………………………………

Thanks for granting me this interview.

For further information contact:

**Dr. MadukaUghasoro (08060388863)**

Dept. of Paediatrics

University of Nigeria Teaching Hospital,

Enugu, Nigeria.
