# Supplementary material for: Acceptability and Willingness-to-Pay for a Hypothetical Ebola Virus Vaccine in Nigeria
Source: PLoS Negl Trop Dis. 2015 Jun 15;9(6):e0003838. doi: 10.1371/journal.pntd.0003838 (PMC4467844; doi:10.1371/journal.pntd.0003838)
Supplement: S1 Table — (DOCX) [file pntd.0003838.s003.docx]

S1 Table Characteristics of the respondents in the two localities.

| Variables | Ilaje (Lagos) (n=293) | Ugba (Abia) (n =289) |
| --- | --- | --- |
| Age (years)  Mean (range)  Gender  Male  Female  Ethnicity  Ibo  Yoruba  Hausa/Fulani  Other Nigerian tribes  Ghanaian/Togolese/Benin Rep.  Religion  Christians  Islam  Traditional  Educational status  No formal  Primary  Secondary  Tertiary  Occupation  Unemployed  Employed in Public sector  Employed in Private sector  Self-employed big business  Self-employed small business  Socioeconomic quartiles  Poorest (Q1)  Very poor (Q2)  Poor (Q3)  Least poor (Q4)  Missing data | 37.7 (26 – 60)  215 (73.3)  78 (26.7)  113 (38.6)  117 (39.9)  39 (13.3)  9 (3.1)  15 (5.1)  202 (69.1)  91 (30.9)  0 (0.0)  0 (0.0)  20 (6.7)  127 (43.5)  146 (49.8)  39 (13.4)  43 (14.8)  75 (25.6)  36 (12.2)  100 (34.0)  73 (24.9)  73 (24.9)  73 (24.9)  74 (25.3)  7 (2.3) | 38.32 (22 – 63)  133 (45.9)  156 (54.1)  280 (97.0)  2 (0.7)  1 (0.3)  6 (2.0)  0 (0.0)  288 (99.7)  1 (0.3)  0  0 (0.0)  40 (13.8)  118 (40.9)  131 (45.3)  10 (3.4)  66 (23.0)  32 (11.0)  78 (27.0)  103 (35.6)  72 (24.9)  72 (24.9)  72 (24.9)  73 (25.3)  11 (3.7) |
