# Supplementary material for: Acceptability and Willingness-to-Pay for a Hypothetical Ebola Virus Vaccine in Nigeria
Source: PLoS Negl Trop Dis. 2015 Jun 15;9(6):e0003838. doi: 10.1371/journal.pntd.0003838 (PMC4467844; doi:10.1371/journal.pntd.0003838)
Supplement: S2 Table — (DOCX) [file pntd.0003838.s004.docx]

S2 Table Discrepancies in WTP for EVV according to different socioeconomic quartiles and household size

| **Variables** | **WTP** | **Mean Maximum amount WTP USD (Naira)** |
| --- | --- | --- |
| **Socioeconomic status**  Q1 (poorest; n = 155)  Q2 (very poor; n= 155)  Q3 (poor; n=155)  Q4 (least poor; n=157)  **Household size**  1-3  4-5  ≥6 | 72.7%  88.1%  90.0%  69.2%  72.7%  83.3%  81.3% | USDI5.0 (2555.6)  USD13.4 (2270.0)  USD 20.5 (3480.0)  USD13.7 (2333.0)  USD17.1 (2905.2)  USD20.7 (3525.0)  USD19.5 (3323.1) |

“n “ is the total number of respondents in each quartile; WTP = Willingness-to-pay.
